# Influence of ferritin levels and inflammatory markers on HbA1c in the Type 2 Diabetes mellitus patients

**DOI:** 10.12669/pjms.35.4.1003

**Published:** 2019

**Authors:** Nazan Erenoglu Son

**Affiliations:** Dr. Nazan Son, Assistant Professor, Afyonkarahisar Saglik Bilimleri University, Afyonkarahisar, Turkey

**Keywords:** Diabetes mellitus, Serum ferritin, C-Reactive Protein (CRP), *Glycated hemoglobin* (HbA1c)

## Abstract

**Objective:**

Diabetes Mellitus (DM) is a significant public health issue worldwide due to the associated comorbidities. Recent studies have demonstrated a strong relationship between blood glucose levels and serum ferritin levels in patients with type 2 DM. The aim of the study was to investigate the association between Ferritin Levels and Inflammatory Markers on HbA1c in the Type 2 Diabetes Mellitus Patients.

**Methods:**

This single-center, cross-sectional, controlled study included patients who were admitted to the Endocrine and Metabolic Disorders outpatient clinics of the Private Kütahya Hospital in the province of Kutahya in the Western Turkey. The study included a total of 172 patients, 84 of whom had type 2 DM and 88 without diabetes and constituted the control group. A total of 190 patients with DM were admitted to the Adult Endocrine and Metabolic Diseases Outpatient Clinics of the hospital between July 1, 2018 and September 1, 2018, and among these, the study was conducted on 172 volunteer patients who met the study inclusion criteria and who did not have any missing data. The HbA1c levels, serum ferritin, hemoglobin (Hb), insulin, Homeostatic Model Assessment of Insulin Resistance (HOMA-IR), C-Reactive Protein (CRP), lipid profiles, and uric acid levels were compared between the groups.

**Results:**

The serum ferritin levels of the patients with type 2 DM significantly increased with increasing HbA1c levels (p<0.01). A strong positive correlation was found between serum ferritin levels and HbA1c and fasting blood glucose (FBG) levels (p<0.01).

**Conclusions:**

Our study results show a significant relationship between HbA1c levels and serum ferritin and CRP levels, suggesting that serum ferritin and CRP levels can be used as a routine screening tool for the early diagnosis of DM. However, further large-scale, prospective studies are needed to confirm these findings.

## INTRODUCTION

Diabetes Mellitus (DM) is one of the most common endocrine disorders around the world, affecting about 366 million people in 2011, although this number is expected to rise to 552 million by the year of 2030.^1^ The early diagnosis and treatment of DM is of particular importance in the prevention comorbidities and mortality. Furthermore, the treatment costs associated with diseases resulting from uncontrolled hyperglycemia in patients with DM constitute a significant burden on the national economies.^2^,^3^ The early diagnosis and treatment of the disease is important in every respect, and in particular, the primary goal of early diagnosis and treatment of type 2 DM associated with obesity is to minimize disease-related risks.

Recent studies into the early recognition of type 2 DM have identified the presence of a significant relationship between serum ferritin levels and Fasting Blood Glucose (FBG) levels in patients with the disease, and these findings have shifted focus on the inflammatory markers of patients with type 2 DM. Normally, serum ferritin levels reflect the status of iron reserves in healthy individuals, and several studies have found increased ferritin levels in association with such diabetic complications as retinopathy, nephropathy, and vascular dysfunction in patients with DM and with elevated FBG.^4^,^5^ A prospective cohort study conducted in China showed a significant correlation between serum ferritin levels and HbA1c and FBG^6^, and epidemiological studies confirmed this correlation.^7^ In their study, Chen et al.^6^ reported excessive iron as a cause of metabolic syndrome, while insulin resistance decreased with decreasing serum ferritin levels^.^ The studies conducted by Kunutsor et al. also supported these findings.^7^ Many studies into this subject, however, have raised the question of whether serum ferritin levels and other inflammatory markers can serve as a marker in the early diagnosis of type 2 DM, although studies into the issue are lacking.

In practice, HbA1c reflects the mean blood glucose levels over the past three months, with an increased HbA1c value (recommended ranges in non-diabetic individuals ≤6)^8^, indicating poor glycemic control. In the present study, we aimed to investigate the relationship between HbA1c levels in patients with type 2 DM and insulin, serum ferritin, C-Reactive Protein (CRP) levels and the levels of certain inflammatory markers.

## METHODS

This single-center, cross-sectional, study included patients who attended the Endocrine and Metabolic Disorders outpatient clinics of the Private Kütahya Hospital in the province of Kutahya in the Western Turkey. The study protocol was approved by the Clinical Trials Ethics Committee of Afyonkarahisar Health Sciences University, Faculty of Medicine (No. 2018/206, Date: 10.09.2018). A written informed consent was obtained from each participant. The study was conducted in accordance with the princibles of the Declaration of Helsinki.

The study included a total of 172 patients, 84 of whom had type 2 DM and 88 without diabetes and constituted the control group. A total of 190 patients with DM were admitted to the Adult Endocrine and Metabolic Diseases Outpatient Clinics of the hospital between July 1, 2018 and September 1, 2018, and among these, the study was conducted on 172 volunteer patients who met the study inclusion criteria and who did not have any missing data. The sociodemographic data were obtained using a face-to-face interview technique. The duration of diabetes in the participating patients with current therapies (i.e., oral antidiabetics, insulin, oral antidiabetic combined with insulin) and comorbidities were evaluated. The serum ferritin levels of the participating patients were carefully reviewed and females with a hemoglobin (Hb) level of ≤12 mg/dl and males with a hemoglobin level of ≤13 mg/dl, those who received therapy for anemia in the last two months, those who donated blood in the last four months, pregnant women, patients with liver or coronary heart disease, those with infections, those receiving any type of radiotherapy or chemotherapy, patients with kidney disease and those with a hematological disease which could affect ferritin levels were excluded from the study. The control group comprised healthy individuals who did not have DM and who did not meet the aforementioned exclusion criteria.

The heights of the participants were measured with a stadiometer (cm), weight was measured with a weighing scale (kg), and blood pressure was measured with a mercury sphygmomanometer by a single operator. The results were recorded. The patients’ Body Mass Index (BMI) values were calculated using the formula of weight (kg) / height (m^2^), and classified as follows: <18.5 underweight, ≥18.5–24.9 normal weight, 25–29.9≤ overweight, and ≥29.9 obese.^9^ The HOMA-IR values, as an indicator of insulin resistance, were calculated using the following formula: FBG (mg/dl) x insulin/ 405,^10^ and a HOMA-IR value ≥2.5 was considered to indicate insulin resistance. Hemoglobin (Hb), hematocrit (Hct), *White Blood Cell* (WBC) count, platelet (PLT), ferritin, vitamin B_12_ (Vit- B_12_), CRP, insulin, HbA1c, creatinine, serum alanine aminotransferase (ALT), FBG, cholesterol, triglyceride (TG), low-density lipoprotein cholesterol (LDL-c), high-density lipoprotein cholesterol (HDL-c), calcium and uric acid (UA) levels in the blood samples collected from the participants following 10 to 12 h of fasting were retrieved from the hospital automation system. The analyses were conducted using the Sysmex XN-1000™ Hematology Analyzer^11^ (Sysmex Group’s, Kobe, Japan) and Roche COBAS Integra 400^12^ devices (Roche Diagnostics Operations, IN, USA). Table-I, II, III shows the classification of HbA1c % levels in patients with type 2 DM, for which the recommendations of the Diabetes Control and Complications Trial (*DCCT*) were considered in the classification. Accordingly, a HbA1c value of 4.0–6.0% was considered normal^8^, a value of 6–7.49% was considered to be within acceptable limits, a value of 7.5–8.99% was considered high, a value of 9–10.49% was considered very high, and a value of ≥10.5% was considered uncontrolled hyperglycemia.

**Table I:** Baseline characteristics of the entire study population.

	Diabetes group (n=84)		Non-diabetes group (n=88)		p value

	Mean± Std. Deviation	Median Min- Max	Mean± Std. Deviation	Median Min- Max	p
Age (years)	59.96±11.26	62.00 (50.25-70.00)	37.68±7.08	38.00 (32.00-41.00)	<0.001
Diabetes years	10.69±7.17	* (5.00-15.00)	-	-	<0.001
SBP(mmHg)	123.57±15.57	120.00 (110.00-130.00)	117.95±7.18	120.00 (115.00-120.00)	0.054
DBP(mmHg)	74.40±8.23	70.00 (70.00-80.00)	77.61±6.99	75.00 (70.00-85.00)	<0.001
BMI(kg/m2)	29.28±6.49	28.43 (24.67-33.26)	22.67±1.48	22.77 (21.50-23.89)	<0.001
Hb(g/dL)	14.17±1.67	14.15 (13.03-15.35)	13.83±1.82	13.90 (12.50-15.40)	* 0.211
Hct(%)	42.16±3.98	41.60 (39.48-44.65)	39.25±4.38	39.30 (36.13-42.55)	* <0.001
PLT(103/µL)	287035.71±83383.49	277500.00 (230000.00-330750.00)	244568.18±53038.44	237000.00 (203250.00-280250.00)	<0.001
WBC(103/µL)	8099.50±2508.94	7840.00 (6492.50-9622.50)	7237.27±1843.83	6950.00 (6025.00-8200.00)	0.006
Ferritin(ng/mL)	81.27±37.20	73.30 (66.25-117.75)	63.69±17.00	65.50 (49.00-78.00)	<0.001
Vit- B_12_ (pg/ml)	444.19±165.37	385.00 (334.25-615.50)	359.41±64.63	350.00 (312.50-390.00)	<0.001
Serum-CRP	7.65±4.92	6.85 (4.10-9.78)	2.77±1.23	2.50 (1.90-3.70)	<0.001
Insülin	8.56±2.07	8.10 (6.90-10.55)	10.02±0.48	10.10 (9.80-10.20)	<0.001
HbA1c	9.09±2.73	8.25 (6.90-10.85)	5.02±0.59	5.20 (4.50-5.50)	<0.001
HOMA-IR	3.76±1.34	3.55 (2.80-4.55)	2.18±0.78	2.08 (1.96-2.25)	<0.001
Kreatinin(mg/dl)	0.78±0.21	0.75 (0.62-0.90)	0.77±0.21	0.75 (0.64-0.90)	0.982
ALT(U/L)	20.02±9.19	17.00 (14.00-24.00)	20.35±8.65	19.00 (14.25-24.75)	0.476
Glukoz(mg/dl)	190.08±91.60	163.50 (130.50-230.25)	87.99±29.95	84.50 (80.00-91.00)	<0.001
Cholosterol(mg/dl)	188.06±37.87	198.00 (168.00-211.00)	185.03±37.36	182.00 (156.00-203.75)	0.174
TG(mg/dl)	266.51±181.24	205.50 (136.25-372.80)	155.60±81.63	140.00 (118.25-163.00)	<0.001
LDL- c(mg/dl)	128.05±47.19	131.50 (101.50-153.00)	118.28±25.59	114.00 (98.00-131.75)	0.056
HDL- c(mg/dl)	47.61±13.63	44.25 (39.00-52.75)	48.76±10.41	49.00 (43.00-56.00)	>0.05
Calcium(mg/dl)	9.34±0.49	9.20 (9.10-9.68)	9.34±0.53	9.10 (9.10-9.78)	0.814
Uric acid(mg/dl)	6.13±1.14	6.50 (5.60-6.90)	4.50±1.03	4.30 (3.70-5.18)	<0.001

* Independent Samples TTest. Mean±Std. Deviation, parametric data’s (Diabetes years-Hb-Hct)

Mann-Whitney Rank Sum Test. Median (25%-75%), non-parametric datas.

**Table II:** Comparison of HbA1c% with biochemical parameters.

		HbA1c	n	Mean Std. Deviation	Median (25%-75%)	p	Multiple Comparisons
Ferritin(ng/mL)	1	6-7.49	33	69.56±27.68	68.00 (56.50-79.00)	<0.001	1-3. 2-4
	2	7.5-8.99	19	67.41±30.10	68.00 (65.00-70.60)		
	3	9-10.49	11	86.73±21.89	89.00 (68.00-101.00)		
	4	10.5 ≥	21	109.37±47.00	128.00 (117.50-136.50)		
Serum-CRP	1	6-7.49	33	5.48±3.68	4.90 (3.65-6.85)	<0.001	1-4. 2-4
	2	7.5-8.99	19	7.39±7.22	5.10 (3.20-6.70)		
	3	9-10.49	11	9.69±2.95	9.60 (8.10-9.90)		
	4	10.5 ≥	21	10.24±3.24	9.80 (8.90-11.20)		
InsÜlin	1	6-7.49	33	10.43±1.38	11.00 (9.50-11.70)	<0.001	1-2. 1-3. 1-4. 2-4
	2	7.5-8.99	19	8.64±1.28	8.30 (7.90-8.90)		
	3	9-10.49	11	7.49±0.77	7.30 (7.00-7.50)		
	4	10.5 ≥	21	6.11±0.44	6.10 (5.75-6.50)		
Glukoz(mg/dl)	1	6-7.49	33	139.05±36.28	141.00 (105.00-171.00)	<0.001	1-4
	2	7.5-8.99	19	178.84±57.23	178.00 (147.00-228.00)		
	3	9-10.49	11	178.18±74.29	158.00 (112.00-231.00)		
	4	10.5 ≥	21	286.67±112.77	280.00 (172.00-365.00)		
TG(mg/dl)	1	6-7.49	33	199.84±106.05	169.00 (141.00-239.00)	<0.001	1-4. 2-4
	2	7.5-8.99	19	224.86±238.44	137.00 (94.10-286.00)		
	3	9-10.49	11	274.36±230.08	174.00 (131.00-389.00)		
	4	10.5 ≥	21	404.86±107.58	389.00 (342.50-464.00)		

One Way Analysis of Variance. Mean± Std. Deviation.

Kruskal-Wallis One Way Analysis of Variance on Ranks. Median (%25-%75)

**Table III:** Correlation of HbA1c% with serum ferritin levels in type 2 DM.

HbA1c range %	Serum Ferritin	

	r value	p value
6-7.49	0.278	0.118
3	-0.011	0.965
4	0.540	0.086
5	-0.362	0.107

Spearman Correlation test.

## Statistical Analysis

Statistical analysis was performed using the SPSS version 21.0 software (IBM Corp, Armonk, NY, USA). Continuous quantitative variables were expressed in number, mean and Standard Deviation (SD); while qualitative or score variables were expressed in number, median, or 25^th^ and 75^th^ percentile Continuous variables of independent measurements showing normal distribution were analyzed using an Independent samples t-test and one-way analysis of variance, while data with abnormal distribution were analyzed using the Mann-Whitney rank sum test and Kruskal-Wallis ANOVA on the ranks test. Variables with abnormal distribution were analyzed using the Spearman’s correlation coefficient to identify the relationship between the variables and the direction. Categorical variables were analyzed using the chi-square test. A *p* value of <0.05 was considered statistically significant.

## RESULTS

A total of 172 patients (84 diabetic, 88 non-diabetic) were followed during the study. The mean duration of diabetes was 10.69±7.17 (range: 5.00 to 15.00) years in the diabetes group. Of the total patients, 65.5% with DM were taking oral antidiabetics, 15.5% were receiving insulin, and 19% were receiving insulin combined with an oral antidiabetic. The patients being followed were divided into two groups, being the diabetes group and the non-diabetic group, and the baseline characteristics of the groups are shown in Table-I.

In the present study, the mean HbA1c value was 9.09±2.73% among the patients with DM, and 5.02±0.59% in the control group. The mean HbA1c % value was significantly higher in the diabetes group than in the non-diabetic group, indicating a significant difference between the groups. Furthermore, an increase was observed in the HbA1c values with prolonged duration of diabetes, indicating that the compliance of patients with therapy decreased and glycemic control worsened with prolonged duration of DM. Apart from the HbA1c % and serum ferritin levels, the BMI, PLT, WBC, Vit B_12_, serum CRP, HOMA-IR, FBG, TG, and UA values of the patients with type 2 DM were significantly higher than the control group. Furthermore, insulin values were significantly higher in the diabetes group than the control group. When the HbA1c % values of the diabetic patients were classified in the present study, the serum ferritin, serum CRP, FBG, and TG values increased significantly and insulin values decreased significantly with increasing HbA1c % values.

The BMI, Hct, PLT, WBC, Ferritin, Vit- B_12_, CRP, HbA1c, HOMA-IR, FBG, TG and UA values of the patients with type 2 DM were significantly higher than those of the non-diabetic patients, and the insulin levels of non-diabetic patients were significantly higher than those of the patients with type 2 DM (Table-I). The HbA1c values of the patients with type 2 DM in the group is shown in Table-II.. A significant increase in the ferritin, CRP, FBG, and TG levels and a significant decrease in insulin levels were observed with worsening glycemic status (Table-II). Table-III shows the correlation between the HbA1c % groups of patients with DM and serum ferritin levels is presented in Table-III. The crossing of groups in the Spearman’s correlation analysis did not show a significant correlation.

## DISCUSSION

There have been many studies carried out worldwide evaluating the relationship between body iron stores and the risk of developing DM. Some of these studies failed to identify a significant relationship between serum ferritin levels and DM.^13^ A study conducted by Elimam et al.^14^ observed significant positive correlations be-tween HbA1c and CRP levels and between HbA1c and serum ferritin levels. All these findings suggest a link between inflammation and glycemic control in patients with T2DM.^14^ On the other hand, there have been various studies suggesting a relationship between elevated body iron stores and serum insulin and blood glucose levels.^15^ Although the results of these studies seem to be inconsistent, a review of literature reveals a higher number of studies suggesting a positive correlation. In a study by Ford et al.^16^ Conducted in 1999, serum ferritin levels were found to be elevated in 9,486 diabetic patients in the United States, while in a study in China conducted by Liu et al.^17^, it was reported that the exact relationship between serum ferritin and the risk of developing type 2 DM was unknown, although an increase in the HOMA-IR values was noted in parallel to the increases in serum ferritin levels and the decreases in insulin secretion from the pancreas.

Furthermore, a study conducted by Wolide et al.^18^ in Ethiopia reported higher ferritin, waist circumference, BMI, and blood pressure values in patients with type 2 DM, and a strongly significant difference to the control group (*p*<0.0001). In a cohort study conducted by Cugy et al.^19^ In France, a significant relationship was reported between serum ferritin levels and elevated liver enzymes and hepatic inflammatory markers (ALT, AST, GGT). In the present study, no significant difference in ALT levels, as one of the hepatic enzymes, was found between the diabetic group and the non-diabetic group. There are also studies suggesting that elevated serum CRP levels are associated with metabolic syndrome. Obese patients, obese patients with type 2 DM, and patients with type 2 DM were compared with a control group in a study conducted to examine the relationship between parameters reflecting body iron stores, and the study found higher serum ferritin and CRP levels among patients than the control group.^20^ This finding suggested that there was a significant relationship between type 2 DM, obesity, and serum ferritin levels. Furthermore, the authors found that elevated CRP levels could be regarded as an indicator of metabolic syndrome. Another study associated ferritin levels with the risk of developing type 2 DM and elevated levels of the markers of inflammation.^21^ Increased serum ferritin and CRP levels are a primary risk factor for the development of chronic diseases. In the present study, serum CRP levels were significantly higher in patients with diabetes than in the non-diabetic patients.

There are also studies comparing two sexes and reporting different opinions and results, although the present study included general population, and also reported no results in this regard. In a study by Dekker et al.^22^ Conducted on ethnic groups, 508 patients from the Netherlands, 597 African Surinamese patients, and 339 South Asian Surinamese patients aged between 35 and 60 years were evaluated. The authors found that serum ferritin levels were positively related with the FBG in patients with type 2 DM, although there were sex differences with a higher positive relationship between the FBG and serum ferritin concentrations in female patients than males among all ethnic groups. A study conducted in China showed a stronger relationship between the serum ferritin levels and diabetes, metabolic syndrome and obesity in male patients than in female patients.^23^ In a cohort study by Chen et al.^6^ Evaluating the relationship between serum ferritin levels and the risk of developing type 2 DM in the Chinese population, 2,225 Chinese subjects aged between 25 and 75 years were evaluated, and among this population, 112 patients with type 2 DM were found. The study made a comparison of diabetics and non-diabetics, and at the end of the study, the baseline serum ferritin levels, BMI, HOMA, blood pressure-hemoglobin HbA1c, cholesterol, HDL-c, ALT, and TG values of the patients with DM were found to be higher than the non-diabetic group. The study also evaluated DM and serum ferritin levels between two sexes and found that serum ferritin levels were associated with a risk of development of DM in Chinese males. Based on these findings, the authors concluded that serum ferritin levels could be used as a biomarker in predicting the risk of developing type 2 DM in males.

In a cross-sectional study including 8,235 participants in 2009 [644 (7.8%) diabetics and 7,591 (92.2%) non-diabetics], the diabetic patients were found to have higher serum ferritin levels, and a significant relationship was found with the HbA1c and HOMA-IR values.^24^ The study concluded that elevated serum ferritin levels could be a marker of risk of developing DM. In a meta-analysis conducted by Kunutsor et al.^7^ 12 of 730 manuscripts related to serum ferritin and DM were analyzed. In this meta-analysis involving 185,462 participants and 11,079 patients with type 2 DM, elevated serum ferritin levels were suggested to be useful in identifying individuals at a high risk for developing type 2 DM.

## CONCLUSION

The results of the present study are consistent with many other studies, and suggest a significant relationship between serum ferritin and glycaemia. However, further large-scale, long-term, prospective studies in different regions of the world are required to gain a better understanding of the role of serum ferritin levels in the prevention of diabetes.

### Authors Contribution

**SN:** The whole article was prepared by me conceived, designed, data collection, manuscript writing designed, statistical analysis, editing, review and final approval of manuscript.
